# *ZNF542P* is a pseudogene associated with LDL response to simvastatin treatment

**DOI:** 10.1038/s41598-018-30859-y

**Published:** 2018-08-20

**Authors:** Kyungpil Kim, Elizabeth Theusch, Yu-Lin Kuang, Andrea Dose, Katrina Mitchel, Celia Cubitt, Yii-Der I. Chen, Ronald M. Krauss, Marisa W. Medina

**Affiliations:** 1 Children’s Hospital Oakland Research Institute, 5700 Martin Luther King Jr Way, Oakland, CA 94609 USA; 2 Los Angeles Biomedical Research Institute at Harbor-UCLA, Torrance, CA 90502 USA

## Abstract

Statins are the most commonly prescribed cardiovascular disease drug, but their inter-individual efficacy varies considerably. Genetic factors uncovered to date have only explained a small proportion of variation in low-density lipoprotein cholesterol (LDLC) lowering. To identify novel markers and determinants of statin response, we used whole transcriptome sequence data collected from simvastatin and control incubated lymphoblastoid cell lines (LCLs) established from participants of the Cholesterol and Pharmacogenetics (CAP) simvastatin clinical trial. We looked for genes whose statin-induced expression changes were most different between LCLs derived from individuals with high versus low plasma LDLC statin response during the CAP trial. We created a classification model of 82 “signature” gene expression changes that distinguished high versus low LDLC statin response. One of the most differentially changing genes was zinc finger protein 542 pseudogene (*ZNF542P*), the signature gene with changes most correlated with statin-induced change in cellular cholesterol ester, an *in vitro* marker of statin response. *ZNF542P* knock-down in a human hepatoma cell line increased intracellular cholesterol ester levels upon simvastatin treatment. Together, these findings imply a role for *ZNF542P* in LDLC response to simvastatin and, importantly, highlight the potential significance of noncoding RNAs as a contributing factor to variation in drug response.

## Introduction

Statins, the most widely prescribed drugs for reducing cardiovascular disease risk, exhibit wide inter-patient variability in their efficacy with regard to the magnitude of plasma low-density lipoprotein cholesterol (LDLC) reduction^1,2^. Genome-wide association studies (GWAS) have identified a modest number of SNPs associated with LDLC statin response in or near genes such as *APOE*, *LPA*, *ABCG2*, *SORT1*, and *SLCO1B1*^3–6^. However, these variants together account for only about 5% of the variance in LDLC statin response^6^.

Recognizing that GWAS have only had limited success in pharmacogenomics, we and others have performed transcriptomic profiling to cumulatively investigate gene expression variation to predict complex drug efficacy^7–10^. For example, using microarray expression data from immortalized lymphoblastoid cell lines (LCLs) derived from participants of the Cholesterol and Pharmacogenetics (CAP) 6 week 40 mg/day simvastatin clinical trial^1^, we recently identified a panel of 100 signature genes whose endogenous expression levels differentiated the high *vs*. low statin LDLC responders^7^. With a radial-basis support vector machine (SVM), we found that these signature genes explained 12.3% of the variance in statin-mediated LDLC change, which is the largest proportion of variance in statin efficacy explained by molecular biomarkers to date. Similarly, Geeleher *et al*. used expression data from untreated tumor cell lines to predict patients’ chemotherapeutic response, demonstrating that that their approach outperformed other existing biomarkers (such as genomic DNA analysis) in several clinical datasets^11^.

Given the success of RNA expression profiling of patient-derived cell lines for the identification of novel drug response genes, here we sought to extend our findings from the CAP LCL expression array studies by using whole transcriptome sequencing (RNA-seq) data from *in vitro* simvastatin versus control incubated cell lines established from 104 European American and 53 African American ancestry CAP participants. RNA-seq can detect thousands of transcripts that are not traditionally present on expression arrays, such as long non-coding RNAs. In addition, unlike our earlier analysis, which was limited to endogenous expression variation from European American ancestry cell lines alone, in this study we attempted to maximize signal by testing for differences in the change in gene expression after simvastatin treatment using RNA-seq data from the tails of the European American LDL response distribution, and testing for cross-ancestry replication in the tails of the African American LCL response distribution. Here we report identification of 82 “signature” genes whose statin-induced expression changes differed between the tails of the LDLC response distribution. From this analysis, zinc finger protein 542 pseudogene (*ZNF542P*) emerged as a novel candidate gene implicated in LDL simvastatin response.

## Results

### Identification of signature genes in cell lines from European American and African American donors

Our goals were to: (i) to identify genes whose simvastatin-induced change in expression levels differed between LCLs derived from high and low LDLC simvastatin responders (Supplementary Fig. S1), and (ii) validate the utility of signature genes in differentiating high and low responders in classification models. For this analysis, we first identified signature genes from LCLs established from the tails of the European American LDLC response distribution, and then we tested for replication in the extremes of the African American LDLC response distribution. The clinical characteristics of these populations are shown in Table 1 and Supplementary Table S1.

**Table 1:** Clinical characteristics of study participants.

	European American		African American	
	High	Low	High	Low
N	25	25	12	14
Men	44%	60%	50%	50%
Smoker (%)	4.0%	8.0%	8.3%	14.3%
Age (yrs)	51.9 ± 14.6	54.5 ± 8.5	54.2 ± 7.8	51.7 ± 12.8
Before treatment LDLC level (mg/dl)	132 ± 36	126 ± 26	129 ± 28	113 ± 32
LDLC percent change after statin treatment (%)	−59.1 ± 3.9%	−22.2 ± 8.0%	−57.5 ± 3.2%	−21.5 ± 6.5%
LDLC level change after statin treatment (mg/dl)	−78.5 ± 23.0	−28.2 ± 11.7	−74.3 ± 16.9	−24.3 ± 10.6

Data are presented as numbers, percentages or means ± s.d.

**Figure 1:**
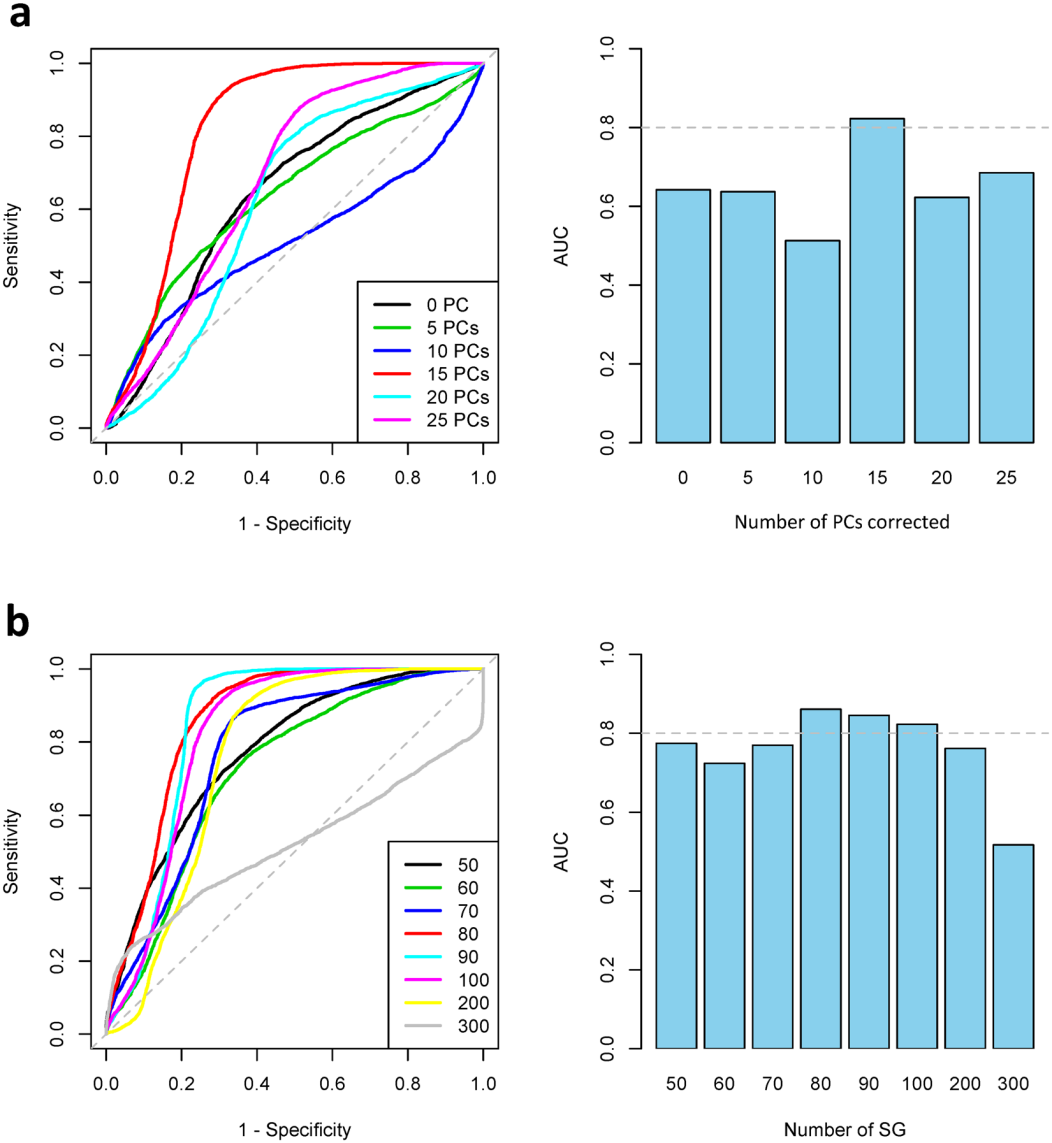
Prediction of statin sensitivity in 12 African American high and 14 African American low responders. Area under the curve (AUC) values were calculated from ROC curves of sensitivity *vs*. 1-specificity as the classification threshold was varied, with (**a**) the top 100 signature genes derived from six different datasets corrected by different numbers of PCs and (**b**) varying numbers of signature genes from the 15 PC corrected dataset.

Gene expression measurements can be strongly influenced by experimental and other confounders that may impede the detection of the desired transcriptomic signal (namely genetically regulated expression differences underlying inter-individual variation in LDLC statin response). To minimize the effects of these confounders, we used a principal components (PCs) based correction method (details in Materials and Methods), where each PC served as a proxy for unknown sources of variation in the data^12,13^. RNA-seq expression changes from both European American and African American LCLs were adjusted by progressively increasing from the 1^st^ to the 25^th^ PC to generate 25 datasets. Next, each dataset was split by ancestry (European American or African American), and 25 each of high and low responders from the European American population (Supplementary Fig. S1a) were used to identify differentially changing genes (signature genes) by empirical Bayes moderated *t*-statistics. Using the expression changes of the identified European American signature genes that were identified as predictors in radial-basis SVM classification models, we then trained and predicted their ability to distinguish 12 high and 14 low African American responders (Supplementary Fig. S1b) whose LDLC statin response was as extreme as the European American subset. A comparison of the prediction performance of the top 100 signature genes demonstrated that, of the different PC corrections, the dataset that corrected for 15 PCs performed best. Notably, a receiver operating characteristic (ROC) analysis using this dataset yielded an area under the curve (AUC) of 0.82 (Fig. 1a).

To evaluate the effects of PC correction, we obtained the first 20 PCs from the 5, 10 and 15 PC corrected datasets, and we tested for correlations with several potential non-genetic covariates (Supplementary Fig. S2). With non-corrected data, most of the covariates showed significant associations with several major PCs. However, these associations became less significant as the number of adjusted PCs increased and were negligible after correction with 15 PCs.

Next, we sought to further refine the model by testing if varying the number of signature genes affected the prediction performance. Using the 15 PC corrected data, we again tested the prediction performance of the European American signature genes in the African American subset, testing varying numbers of the most differentially changing genes. From this analysis, we found that a dataset including the top 80 most differentially changing genes outperformed (AUC = 0.86) other gene sets with smaller or larger numbers of genes (Fig. 1b). To further refine the number of signature genes, we tested additional signature gene sets from 70 to 90 signature genes with a step size of 1 and observed the maximum AUC of 0.88 with 82 signature genes (Supplementary Fig. S3**;** Supplementary Table S2). Of the 82 signature genes, 12 had p-values less than 0.0002 (Table 2), the smallest empirical p-value that could be measured with 5000 permutations, including zinc finger protein 542, pseudogene (*ZNF542P*).

**Table 2:** Top 12 genes most differentially changing between 25 high and 25 low European American LDLC responders.

Ensembl ID	Gene Symbol
ENSG00000105220	*GPI*
ENSG00000114737	*CISH*
ENSG00000118762	*PKD2*
ENSG00000119396	*RAB14*
ENSG00000123595	*RAB9A*
ENSG00000134333	*LDHA*
ENSG00000137766	*UNC13C*
ENSG00000178397	*FAM220A*
ENSG00000182397	*DNM1P46*
ENSG00000215158	*AC138409.2*
ENSG00000240225	*ZNF542P*
ENSG00000263335	*AF001548.2*

All had empirical Bayes moderated t-statistics p-values less than 0.0002 using 5000 permutations.

### Change in intracellular cholesterol ester differs between high and low LDLC responders

**Figure 2:**
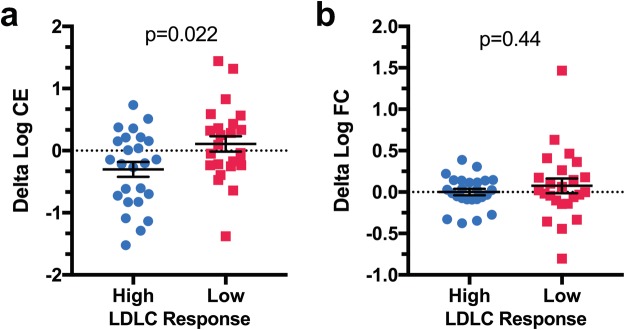
Cellular cholesterol ester statin response differs between LCLs from high versus low LDLC statin responders. Intracellular cholesterol esters (CE, panel a) and free cholesterol (FC, panel b) were measured in LCLs from 25 European American high and 24 European American low statin responders after 24 hr incubation with 2 μM simvastatin or control buffer. Two-sided Student’s T-tests were performed to identify significant differences between high and low responders. Individual data points with mean ± s.e.m. are shown.

Statins inhibit HMGCR, the rate-limiting enzyme in the cholesterol synthesis pathway, leading to reduced intracellular cholesterol levels. This stimulates LDL receptor expression, resulting in increased plasma clearance of LDL and lower plasma LDL levels. Thus, we hypothesized that LCLs from individuals with high LDLC simvastatin response would have greater statin-induced reductions in cellular cholesterol levels compared to LCLs from individuals with low LDLC simvastatin response. Consistent with this hypothesis, we found that *in vitro* simvastatin exposure reduced cellular cholesterol ester levels in LCLs from the high responders, whereas there was either no change or slightly increased cellular cholesterol ester in LCLs from the low responders (Fig. 2a). There was no difference in simvastatin-induced change in free cellular cholesterol levels between high and low responders (Fig. 2b), a finding which was not unexpected given the fact that free cholesterol levels are tightly regulated to maintain homeostasis.

### Identification of *ZNF542P* as a candidate gene

**Figure 3:**
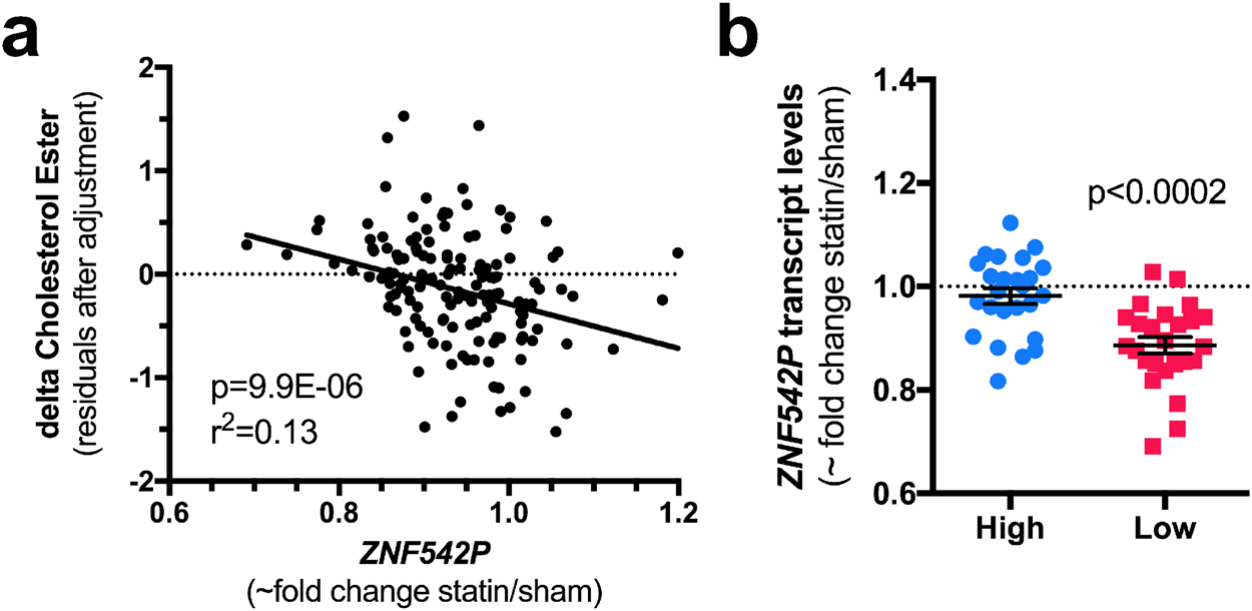
*ZNF542P* statin response is negatively correlated with intracellular cholesterol ester and plasma LDLC statin responses. Statin-induced changes in *ZNF542P* expression levels were quantified in 104 European American CAP LCLs after *in vitro* exposure to 2 μM simvastatin or control buffer. (**a)** *ZNF542P* expression changes were tested for correlation with statin induced change in cellular cholesterol ester (CE) quantified in the statin and control treated LCLs from 103 European Americans. Delta cholesterol ester was calculated as the log difference of the statin minus control treated cells. (**b)** *ZNF542P* expression levels were quantified by RNA-seq in CAP LCLs from 25 European American LDLC high responders and 25 European American LDLC low responders after 24 hr *in vitro* incubation with 2 μM simvastatin or control buffer. P-values were calculated using empirical Bayes moderated t-statistics with 5000 permutations to indicate differences in *ZNF542P* expression changes between high and low responders. For graphical purposes, *ZNF542P* fold change was estimated as 2^(variance stabilized control-variance stabilized statin) since variance stabilization is roughly a log2 transformation, but the statistics were performed on the variance stabilized delta data after correction for 15 PCs. Individual data points with mean ± s.e.m. are shown.

Next, we tested if simvastatin-induced expression differences of any the 82 signature genes were associated with variation in simvastatin-induced change in cellular cholesterol ester. From this analysis, we found that *ZNF542P* was the only signature gene whose change was significantly correlated with cholesterol ester change after correction for multiple testing (Supplementary Table S3). Changes in levels of cholesterol ester were negatively correlated with *ZNF542P* expression change in LCLs from 103 European American donors (Spearman’s rho = −0.348, Bonferroni-adjusted *P* = 0.028, Fig. 3a). Change in levels of free cholesterol change showed no significant association with *ZNF542P* (*P* > 0.6). Not unexpectedly, when change in plasma LDLC was treated as a continuous phenotype, *ZNF542P* expression change adjusted for 15 PCs in LCLs was also correlated with change in LDLC in the plasma during the CAP clinical trial (*P* = 2.35 × 10^−4^, Spearman’s ρ = −0.353, N = 104). A similar trend was observed with change in plasma total cholesterol (Spearman’s ρ = −0.206, *P* = 0.036, N = 104). No significant associations were observed with change in plasma triglycerides or HDLC (*P* > 0.3).

As described above, *ZNF542P* was among the signature genes whose expression in response to simvastatin showed the greatest difference between the European American high and low responders. This was due to reduced expression levels in the low responders, with no effect in the high responders (Fig. 3b).

### *ZNF542P* knock-down increases intracellular cholesterol ester upon statin treatment

**Figure 4:**
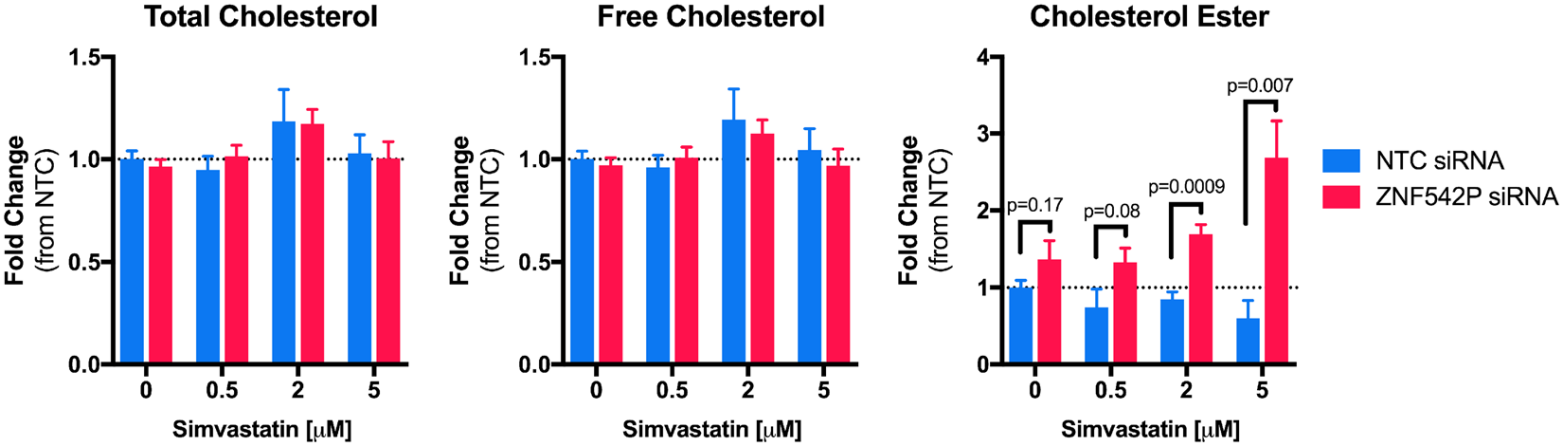
*ZNF542P* knock-down alters cellular cholesterol levels. Huh7 cells were reverse transfected with siRNAs targeting *ZNF542P* or a non-targeting control (NTC), exposed to simvastatin (0.5, 2 or 5 μM) or control buffer for 24 hours, after which cellular cholesterol was quantified, n = 4–12 per treatment condition. Values were normalized to the average of the NTC siRNA control treated cells and shown as mean ± s.e.m.

Since *ZNF542P* has no known function, we tested if knock-down of *ZNF542P* altered intracellular cholesterol levels. Using an siRNA targeting *ZNF542P*, we achieved > 80% knock-down in the Huh7 human hepatoma cell line, Supplementary Figure S4. Under endogenous conditions, we observed a non-statistically significant trend of increased cellular cholesterol ester upon *ZNF542P* knock-down. Importantly, this increase became statistically significant when the cells were exposed to increasing concentrations of simvastatin, with a dose dependent effect observed (Fig. 4). In cells incubated with 5 μM simvastatin, ZNF542P knock-down increased cholesterol ester (2.7 ± 0.48 fold mean ± s.e.m., p = 0.007), in contrast with the reduced cellular cholesterol ester observed in the NTC siRNA treated cells (0.60 ± 0.22 fold mean ± s.e.m., p = 0.03). *ZNF542P* knock-down did not alter intracellular total or free cholesterol under simvastatin or control treated conditions. In addition, *ZNF542P* knock-down did not alter transcript levels of genes we tested involved in cholesterol synthesis (*HMGCR, MVK* and *HMGCS1)* or uptake *(LDLR)* in either simvastatin or control treated conditions, Supplementary Figure S4.

## Discussion

In 2013 the American Heart Association and American College of Cardiology released expanded guidelines for statin prescription, dramatically increasing the number of potential statin users^14,15^. Since statins are not uniformly effective in preventing cardiovascular disease^14^, and the incidence of adverse effects such as myopathy and new-onset diabetes may be more prevalent than originally thought^16–18^, identification of predictors of statin efficacy has become increasingly more important from a public health perspective. To obtain a more comprehensive understanding of genetic markers contributing to variation in simvastatin LDLC response, we applied a radial-basis SVM classification model to whole transcriptome sequence data and identified a set of 82 signature genes whose simvastatin-induced change in expression levels could be used to differentiate between individuals with “high” vs. “low” LDLC response to statin treatment. *ZNF542P* emerged as an interesting novel candidate gene based on two factors: (1) it was one of the signature genes whose expression levels were most differentially changed between the high and low LDLC simvastatin responders, and (2) its simvastatin-induced expression difference was highly correlated to change in cellular cholesterol ester content.

Here, we report that changes in expression of the putative pseudogene *ZNF542P* in simvastatin-exposed LCLs were significantly associated with both changes in cellular cholesterol ester content and with *in vivo* statin-induced changes in plasma LDLC and total cholesterol in the individuals from whom the LCLs were derived. Notably, these relationships were consistent with each other, as greater simvastatin-induced reductions in *ZNF542P* were correlated with smaller reductions in both cellular cholesterol ester content and plasma LDL cholesterol. Furthermore, these results are consistent with our findings that *ZNF542P* knock-down increased cholesterol ester levels upon simvastatin treatment. Cholesterol ester is a storage form of cholesterol that is found primarily within lipid droplets. The lack of effect of *ZNF542P* knock-down on cellular unesterified cholesterol content is not surprising given that this form of cholesterol is located primarily in cell membranes and is subject to tight homeostatic regulation.

To date, there have been no reports regarding the function of *ZNF542P*. Pseudogenes were once thought to be inactive gene sequences, evolutionary remnants from past gene duplication events. However, more recently, several reports have found that long non-coding RNAs (lncRNAs) transcribed from pseudogenes have functional effects on a variety of cellular processes, and they often regulate their protein-coding counterparts^19^. For example, *PTENpg1*, a *PTEN* pseudogene, regulates *PTEN* transcript levels by preventing miRNAs from targeting the *PTEN* transcript^20^. Flanked by *ZSCAN5A* (zinc finger and SCAN domain containing 5A) and *ZNF582* (zinc finger protein 582), *ZNF542P* lies within a cluster of several ZNF genes that have been implicated in gene transcription. However, further study is necessary to determine the mechanism by which *ZNF542P* impacts intracellular cholesterol ester in the context of simvastatin treatment.

Although our findings implicate *ZNF542P* as a novel modulator of LDLC response to simvastatin treatment, other genes identified within the set of 82 signature genes also likely contribute to response. For example, the signature gene set contains solute carrier organic anion transporter family member 2B1 (*SLCO2B1*), a known statin transporter^21^. A SNP in *SLCO2B1* has been implicated in simvastatin pharmacokinetics^22^. Thus, the relationship between the signature gene set expression changes and statin response may be driven by both pharmacodynamic and pharmacokinetic mechanisms.

In summary, we here identify a set of 82 signature genes whose simvastatin-induced change in expression levels distinguish high versus low LDLC statin responders in both European American and African American populations. This gene set contains 13 noncoding RNAs, with *ZNF542P* specifically emerging as a novel candidate gene implicated in cholesterol metabolism and simvastatin response. A recent comprehensive survey of the human transcriptome identified over 91,000 expressed genes, of which 68% are classified as lncRNAs^23^. Our findings highlight the need to further our understanding of the roles of these genes in mediating cellular processes well as determinants of drug response.

## Methods

### Participants and clinical measures

The Cholesterol and Pharmacogenetics (CAP) clinical trial was comprised of 944 participants (609 self-identified whites and 335 self-identified blacks) treated with 40 mg/day simvastatin for 6 weeks (Clinical Trials.gov identifier NCT00451828)^1^. Plasma lipid measures were quantified twice before statin treatment, and after 4 and 6 weeks on-treatment, with delta LDL calculated as the difference of the average pre-treatment vs. the average post-treatment values. All experimental protocols were approved by the institutional review boards at Children’s Hospital Oakland Research Institute and at UCLA and UCSF, where the clinical trial was performed. Lymphoblastoid cell lines (LCLs) were generated from each study participant as previously described^24^. Informed consent for participation in the statin clinical trial and the use of cell lines was obtained from each study subject. All methods were performed in accordance with the relevant guidelines and regulations.

### RNA-seq data generation and analysis

LCL lines from CAP participants were exposed to 2 µM activated simvastatin (provided by Merck Inc., Whitehouse Station, NJ) or control buffer for 24 hours and total RNA was extracted as previously described^10^. Indexed, strand-specific, paired-end Illumina sequencing libraries were prepared by LabCorp (formerly Covance, Seattle, WA) as previously described^10^. Sequences were aligned using TopHat2^25^ and adjusted for library size and variance stabilized using DESeq2^26^ as previously described^10^. Quality control checks were performed as previously described^10^, except that 7 samples in experimental batch 1 were included here but excluded previously.

### RNA-seq expression normalization

Differential RNA-seq expression was determined by subtracting the control-treated variance stabilized data from the statin-treated variance stabilized data and the resulting expression changes for each gene were quantile normalized and adjusted for experiment batch 1 using regression. To adjust expression differences for unmeasured confounders, principal component analysis (PCA) was adopted as previously described^12,13^. Among the PCs of a covariance matrix between samples sorted by the proportion of explained variation in the original matrix, up to 25 PCs were selected such that adding another PC would explain less than 0.5% of the variation. PCs 1 through 25 were progressively regressed out, and the residuals from each regression were quantile normalized and used as the change in expression level of each gene.

### Identifying signature genes

To identify signature genes from 25 each of high and low European American responders, we used empirical Bayes moderated *t*-statistics as in Kim *et al*.^7^. Starting from a *relative difference d*(*i*) which is defined as

1 $$d(i)=\frac{{\bar{x}}_{H}(i)-{\bar{x}}_{L}(i)}{s(i)+{s}_{0}},$$

where ${\bar{x}}_{H}(i)$ and ${\bar{x}}_{L}(i)$ are defined as the average level of expression for gene *i* in the high (*H*) and low (*L*) responder groups, respectively, and *s*(*i*) is the standard deviation of repeated measurements, we introduced varying *s*_0_ values in the denominator to stabilize the variance of *d*(*i*) irrespective of the gene expression level using an empirical Bayes approach. A more detailed description on this method has been previously reported^7^.

### Cross-ancestry prediction using SVM based classification

Radial-basis SVMs were used for training and predicting 12 high and 14 low African American responders in the SVM classification models. The performance of the models was evaluated by randomly splitting the data into 10 sets, with 9 assigned as the training set and the tenth as the testing set. The model was trained using the training set and applied to the testing set for prediction. This process was repeated 5000 times and the prediction power of the model was estimated based on the 5000 testing sets. The SVM function in the R package (kernlab) was used to implement the models with default parameter settings^27^. For the SVM, the radial basis kernel was chosen due to its superior performance in the cross-validation results. The prediction performance was evaluated by ROC curve analysis and quantitated by AUC using the ROCR package in R^28^.

### *in vivo* and *in vitro* association analysis

To measure statin-induced changes of *in vivo* clinical phenotypes for correlation analyses, delta log measures were calculated as the log (average value of each phenotype on treatment) minus the log (average of value of each phenotype two pre-treatment). The distribution of the plasma LDLC change adjusted for age, race and smoking status is shown in Supplementary Figure S1. Plasma HDLC change was adjusted for race.

For *in vitro* cholesterol measurements, lipids were extracted from the CAP LCLs with hexane and isopropyl alcohol (3:2, v/v), and dried under nitrogen. Intracellular total cholesterol and free cholesterol levels were quantified using the Amplex Red Cholesterol Assay Kit (Life Technologies) following manufacturer’s instructions, and normalized to total cellular protein content. For measurement of cholesterol esters, extracted lipids were incubated with esterase up to 2hrs, and cholesterol ester was calculated as difference of the total minus free cholesterol. The change in cholesterol ester was calculated as the delta log of the cholesterol ester in the statin minus control treated cells.

All *in vivo* and *in vitro* phenotypes were tested for association with the *ZNF542P* expression changes using Spearman rank correlation in R.

### Functional studies of *ZNF542P*

Huh7 cells grown in MEM with 10% FBS were reverse transfected with a Silence Select siRNA (Life Technologies) targeting *ZNF542P* (n258919) or a non-targeting control (NTC, assay number AM6411, Life Technologies) using the siPORT transfection reagent as previously described^29^. After 24 hrs, cell culture media was replaced with media supplemented with either 2 μM activated simvastatin or control buffer. Simvastatin was kindly provided by Merck. RNA was extracted using Qiashredders (Qiagen) and the PureLink RNA Mini Kit (Life Technologies), and cDNA was synthesized using the cDNA Archive Kit (Life Technologies). *ZNF542P* values were quantified by a TaqMan assay (n258919_asy, Life Technologies) and normalized to *CLTPM* as a loading control. *HMGCR, HMGCS, MVK* and *LDLR* transcript levels were quantified as previously described^30^. All qPCR reactions were performed in triplicate. Intracellular total cholesterol, free cholesterol, and cholesterol ester were quantified as above. All cultures were verified to be mycoplasma free using the MycoSensor qPCR Assay Kit (Agilent).

One-way ANOVA was used to identify statistically significant effects of *ZNF542P* knock-down on levels of cellular cholesterol and transcripts. For ANOVA p < 0.05, statistically significantly differences between treatment conditions were identified using Tukey’s multiple comparisons test with adjusted p-values reported.

## Electronic supplementary material

Supplementary Information

## Data Availability

RNA-seq and clinical phenotype data used in this analysis are available from dbGaP (phs000481.v2.p1). All other datasets analyzed during the current study are available from the corresponding author on reasonable request.
