# Supplementary material for: ZNF542P is a pseudogene associated with LDL response to simvastatin treatment
Source: Sci Rep. 2018 Aug 20;8:12443. doi: 10.1038/s41598-018-30859-y (PMC6102286; doi:10.1038/s41598-018-30859-y)
Supplement: Supplementary file 1 — Supplementary Information [file 41598_2018_30859_MOESM1_ESM.pdf]

SUPPLEMENTARY MATERIALS

ZNF542P is a pseudogene associated with LDL response to simvastatin treatment

Kyungpil Kim[†1], Elizabeth Theusch[†1], Yu-Lin Kuang[†1], Andrea Dose[1], Katrina Mitchel[1], Celia Cubitt[1], Yii-Der I. Chen[2], Ronald M. Krauss[1], Marisa W. Medina[§1]

	European Americans	African Americans	*p*-value
N	104	53	
Men	60%	53%	0.424
Smoker (%)	8.70%	13.20%	0.406
Age (yrs)	54.2 ± 12.3	51.7 ± 12.5	0.242
Before treatment plasma LDLC level (mg/dl)	134 ± 32	129 ± 37	0.423
LDLC % change with statin (%)	-42.4 ± 14.7%	-39.8 ± 14.6%	0.282
LDLC change with statin (mg/dl)	-57.4 ± 25.6	-52.3 ± 25.7	0.245

Table S1. Clinical characteristics of CAP study participants with RNA-seq data. Data are presented as numbers, percentages or means ± SDs. None of these metrics were significantly different between the ancestry subsets as evaluated with two-tailed Student's t-tests.

SG#	Ensembl ID	Gene Name	P	~HR Fold Change	~LR Fold Change	Non-Protein Coding
1	ENSG00000105220	*GPI*	<0.0002	0.96	0.97	
2	ENSG00000114737	*CISH*	<0.0002	0.96	1.03	
3	ENSG00000118762	*PKD2*	<0.0002	0.96	0.98	
4	ENSG00000119396	*RAB14*	<0.0002	1.01	1.00	
5	ENSG00000123595	*RAB9A*	<0.0002	1.10	1.05	
6	ENSG00000134333	*LDHA*	<0.0002	0.94	0.89	
7	ENSG00000137766	*UNC13C*	<0.0002	1.03	1.06	
8	ENSG00000178397	*FAM220A*	<0.0002	1.05	0.95	
9	ENSG00000182397	*DNM1P46*	<0.0002	1.04	0.98	Y
10	ENSG00000215158	*AC138409.2*	<0.0002	0.95	0.99	Y
11	ENSG00000240225	*ZNF542P*	<0.0002	0.98	0.88	Y
12	ENSG00000263335	*AF001548.2*	<0.0002	1.05	0.99	Y
13	ENSG00000113761	*ZNF346*	0.0002	0.95	0.98	
14	ENSG00000133874	*RNF122*	0.0002	1.09	1.06	
15	ENSG00000143324	*XPR1*	0.0002	1.00	0.95	
16	ENSG00000148429	*USP6NL*	0.0002	1.02	0.97	
17	ENSG00000154102	*C16orf74*	0.0002	0.98	0.96	
18	ENSG00000162298	*SYVN1*	0.0002	1.03	1.10	
19	ENSG00000173409	*ARV1*	0.0002	0.96	0.97	
20	ENSG00000174194	*AGAP8*	0.0002	0.97	1.03	
21	ENSG00000175197	*DDIT3*	0.0002	1.00	0.95	
22	ENSG00000184983	*NDUFA6*	0.0002	1.02	1.05	
23	ENSG00000244733	*AL132656.2*	0.0002	0.97	1.03	Y
24	ENSG00000102144	*PGK1*	0.0004	0.96	0.93	
25	ENSG00000114767	*RRP9*	0.0004	0.93	0.91	
26	ENSG00000160194	*NDUFV3*	0.0004	0.98	1.01	
27	ENSG00000165275	*TRMT10B*	0.0004	1.06	0.98	
28	ENSG00000166689	*PLEKHA7*	0.0004	1.02	0.96	
29	ENSG00000188282	*RUFY4*	0.0004	0.95	1.02	
30	ENSG00000188373	*C10orf99*	0.0004	1.04	0.99	
31	ENSG00000227543	*SPAG5-AS1*	0.0004	0.97	1.01	Y
32	ENSG00000001629	*ANKIB1*	0.0006	1.02	1.02	
33	ENSG00000033170	*FUT8*	0.0006	1.04	1.05	
34	ENSG00000149658	*YTHDF1*	0.0006	0.99	0.96	
35	ENSG00000163704	*PRRT3*	0.0006	1.03	1.13	
36	ENSG00000197841	*ZNF181*	0.0006	1.08	0.96	
37	ENSG00000245112	*SMARCA5-AS1*	0.0006	0.97	1.03	Y
38	ENSG00000065989	*PDE4A*	0.0008	1.02	1.02	
39	ENSG00000111669	*TPI1*	0.0008	0.97	0.96	
40	ENSG00000124279	*FASTKD3*	0.0008	1.00	0.91	
41	ENSG00000137491	*SLCO2B1*	0.0008	0.97	0.94	
42	ENSG00000156515	*HK1*	0.0008	0.96	0.95	
43	ENSG00000159314	*ARHGAP27*	0.0008	0.98	1.03	
44	ENSG00000168282	*MGAT2*	0.0008	1.07	1.09	
45	ENSG00000171208	*NETO2*	0.0008	1.00	1.02	
46	ENSG00000108821	*COL1A1*	0.0010	1.05	1.04	
47	ENSG00000119446	*RBM18*	0.0010	0.99	0.92	
48	ENSG00000198960	*ARMCX6*	0.0010	1.05	1.04	
49	ENSG00000214135	*AC132008.2*	0.0010	0.99	1.03	Y
50	ENSG00000229980	*TOB1-AS1*	0.0010	1.02	1.09	Y

51	ENSG00000244026	*FAM86DP*	0.0010	1.01	0.95	Y
52	ENSG00000107521	*HPS1*	0.0012	1.01	1.03	
53	ENSG00000154781	*CCDC174*	0.0012	1.03	0.96	
54	ENSG00000177683	*THAP5*	0.0012	0.95	0.92	
55	ENSG00000242396	*AC096536.3*	0.0012	1.07	1.02	Y
56	ENSG00000072840	*EVC*	0.0014	1.07	1.05	
57	ENSG00000125967	*NECAB3*	0.0014	0.99	1.07	
58	ENSG00000140961	*OSGIN1*	0.0014	1.04	0.99	
59	ENSG00000151490	*PTPRO*	0.0014	1.11	1.03	
60	ENSG00000164011	*ZNF691*	0.0014	1.05	0.97	
61	ENSG00000186020	*ZNF529*	0.0014	0.99	0.92	
62	ENSG00000114738	*MAPKAPK3*	0.0016	0.97	0.95	
63	ENSG00000135457	*TFCP2*	0.0016	1.03	0.99	
64	ENSG00000157514	*TSC22D3*	0.0016	1.83	1.76	
65	ENSG00000159079	*CFAP298*	0.0016	0.98	0.93	
66	ENSG00000114268	*PFKFB4*	0.0018	1.11	1.05	
67	ENSG00000143702	*CEP170*	0.0018	1.04	0.95	
68	ENSG00000160703	*NLRX1*	0.0018	1.00	1.06	
69	ENSG00000166439	*RNF169*	0.0018	1.04	0.97	
70	ENSG00000187123	*LYPD6*	0.0018	0.98	1.03	
71	ENSG00000213593	*TMX2*	0.0018	0.98	0.95	
72	ENSG00000253626	*EIF5AL1*	0.0018	0.95	1.01	
73	ENSG00000086619	*ERO1B*	0.0020	0.94	0.93	
74	ENSG00000095787	*WAC*	0.0020	1.01	0.97	
75	ENSG00000108651	*UTP6*	0.0020	0.98	0.93	
76	ENSG00000118276	*B4GALT6*	0.0020	1.04	1.03	
77	ENSG00000132953	*XPO4*	0.0020	0.94	0.87	
78	ENSG00000170215	*FAM27B*	0.0020	1.00	0.95	
79	ENSG00000174891	*RSRC1*	0.0020	0.99	1.00	
80	ENSG00000197119	*SLC25A29*	0.0020	1.00	1.10	
81	ENSG00000224220	*AC104699.1*	0.0020	1.03	0.97	Y
82	ENSG00000235428	*WI2-2998D17.2*	0.0020	1.03	1.00	Y

Table S2. List of 82 signature genes that distinguish high versus low LDLC response to statin treatment. Expression levels were quantified by RNA-seq in CAP LCLs from European Americans (25 high and 25 low responders) after 24hr *in vitro* incubation with 2µM simvastatin or sham buffer. Expression fold changes were estimated as $2^{(\text{variance stabilized statin-variance stabilized sham})}$. P-values were calculated from the residuals of the 15 PC adjusted variance stabilized delta data, and indicate difference in statin-induced change in gene expression between high versus low responders. 13 of the 82 SG were non-protein coding according to the Ensemblv67 annotation.

SG#	Ensembl ID	Gene Name	rho	p
11	ENSG00000240225	*ZNF542P*	-0.348	0.00034
20	ENSG00000174194	*AGAP8*	0.265	0.00705
49	ENSG00000214135	*AC132008.2*	0.210	0.03363
6	ENSG00000134333	*LDHA*	-0.201	0.04183
17	ENSG00000154102	*C16orf74*	0.187	0.05897
29	ENSG00000188282	*RUFY4*	-0.186	0.06007
45	ENSG00000171208	*NETO2*	0.180	0.06967
52	ENSG00000107521	*HPS1*	-0.177	0.07371
81	ENSG00000224220	*AC104699.1*	-0.174	0.07922
48	ENSG00000198960	*ARMCX6*	-0.171	0.08394
13	ENSG00000113761	*ZNF346*	0.168	0.08970
1	ENSG00000105220	*GPI*	-0.163	0.10034
53	ENSG00000154781	*CCDC174*	-0.159	0.10923
26	ENSG00000160194	*NDUFV3*	0.153	0.12263
4	ENSG00000119396	*RAB14*	0.150	0.13079
51	ENSG00000244026	*FAM86DP*	-0.148	0.13506
12	ENSG00000263335	*AF001548.2*	-0.142	0.15245
18	ENSG00000162298	*SYVN1*	0.139	0.16051
24	ENSG00000102144	*PGK1*	-0.139	0.16264
2	ENSG00000114737	*CISH*	0.137	0.16650
28	ENSG00000166689	*PLEKHA7*	-0.135	0.17305
3	ENSG00000118762	*PKD2*	0.134	0.17735
47	ENSG00000119446	*RBM18*	-0.133	0.18157
36	ENSG00000197841	*ZNF181*	-0.131	0.18520
71	ENSG00000213593	*TMX2*	-0.130	0.19035
57	ENSG00000125967	*NECAB3*	0.128	0.19603
32	ENSG00000001629	*ANKIB1*	-0.127	0.20054
25	ENSG00000114767	*RRP9*	-0.126	0.20289
10	ENSG00000215158	*AC138409.2*	0.124	0.21070
54	ENSG00000177683	*THAP5*	0.124	0.21224
55	ENSG00000242396	*AC096536.3*	-0.123	0.21413
75	ENSG00000108651	*UTP6*	-0.120	0.22575
40	ENSG00000124279	*FASTKD3*	-0.119	0.22944
27	ENSG00000165275	*TRMT10B*	-0.116	0.24150
33	ENSG00000033170	*FUT8*	0.116	0.24155
37	ENSG00000245112	*SMARCA5-AS1*	0.111	0.26186
7	ENSG00000137766	*UNC13C*	0.109	0.27200
8	ENSG00000178397	*FAM220A*	-0.108	0.27776
67	ENSG00000143702	*CEP170*	-0.103	0.30234
9	ENSG00000182397	*DNM1P46*	-0.102	0.30349
19	ENSG00000173409	*ARV1*	0.102	0.30647
74	ENSG00000095787	*WAC*	0.102	0.30700
34	ENSG00000149658	*YTHDF1*	-0.088	0.37570
63	ENSG00000135457	*TFCP2*	-0.086	0.38460
69	ENSG00000166439	*RNF169*	-0.086	0.38727
44	ENSG00000168282	*MGAT2*	0.085	0.39093
62	ENSG00000114738	*MAPKAPK3*	0.079	0.42582
65	ENSG00000159079	*CFAP298*	0.076	0.44280
64	ENSG00000157514	*TSC22D3*	-0.074	0.45552
14	ENSG00000133874	*RNF122*	-0.068	0.49414
5	ENSG00000123595	*RAB9A*	-0.068	0.49449
39	ENSG00000111669	*TPI1*	-0.068	0.49519
23	ENSG00000244733	*AL132656.2*	0.068	0.49729
61	ENSG00000186020	*ZNF529*	-0.059	0.55624
73	ENSG00000086619	*ERO1B*	0.057	0.56981
76	ENSG00000118276	*B4GALT6*	-0.054	0.59039
72	ENSG00000253626	*EIF5AL1*	0.053	0.59619

82	ENSG00000235428	*WI2-2998D17.2*	-0.052	0.60264
68	ENSG00000160703	*NLRX1*	0.049	0.62155
16	ENSG00000148429	*USP6NL*	-0.046	0.64411
15	ENSG00000143324	*XPR1*	-0.043	0.66893
43	ENSG00000159314	*ARHGAP27*	0.041	0.68039
35	ENSG00000163704	*PRRT3*	-0.040	0.69078
78	ENSG00000170215	*FAM27B*	-0.038	0.69991
50	ENSG00000229980	*TOB1-AS1*	-0.032	0.74664
79	ENSG00000174891	*RSRC1*	0.031	0.75199
77	ENSG00000132953	*XPO4*	-0.029	0.76720
22	ENSG00000184983	*NDUFA6*	-0.029	0.77361
42	ENSG00000156515	*HK1*	-0.027	0.78258
46	ENSG00000108821	*COL1A1*	-0.026	0.79337
30	ENSG00000188373	*C10orf99*	0.025	0.80044
70	ENSG00000187123	*LYPD6*	-0.022	0.82483
56	ENSG00000072840	*EVC*	-0.021	0.83007
58	ENSG00000140961	*OSGIN1*	0.019	0.84922
41	ENSG00000137491	*SLCO2B1*	0.016	0.87010
31	ENSG00000227543	*SPAG5-AS1*	-0.015	0.88193
60	ENSG00000164011	*ZNF691*	-0.011	0.90889
59	ENSG00000151490	*PTPRO*	0.008	0.93718
38	ENSG00000065989	*PDE4A*	-0.007	0.94166
66	ENSG00000114268	*PFKFB4*	0.005	0.95905
80	ENSG00000197119	*SLC25A29*	-0.004	0.96688
21	ENSG00000175197	*DDIT3*	-0.001	0.99371

Table S3. Association of 82 signature gene expression changes with cholesterol ester changes in 103 European American CAP LCLs. Gene expression changes were adjusted for 15 PCs and tested for correlation with delta log cholesterol ester using Spearman's correlation. After adjusting for 82 tests using a Bonferroni correction, *ZNF542P* had an adjusted p-value of 0.028 and *AGAP8* had an adjusted p-value of 0.578.

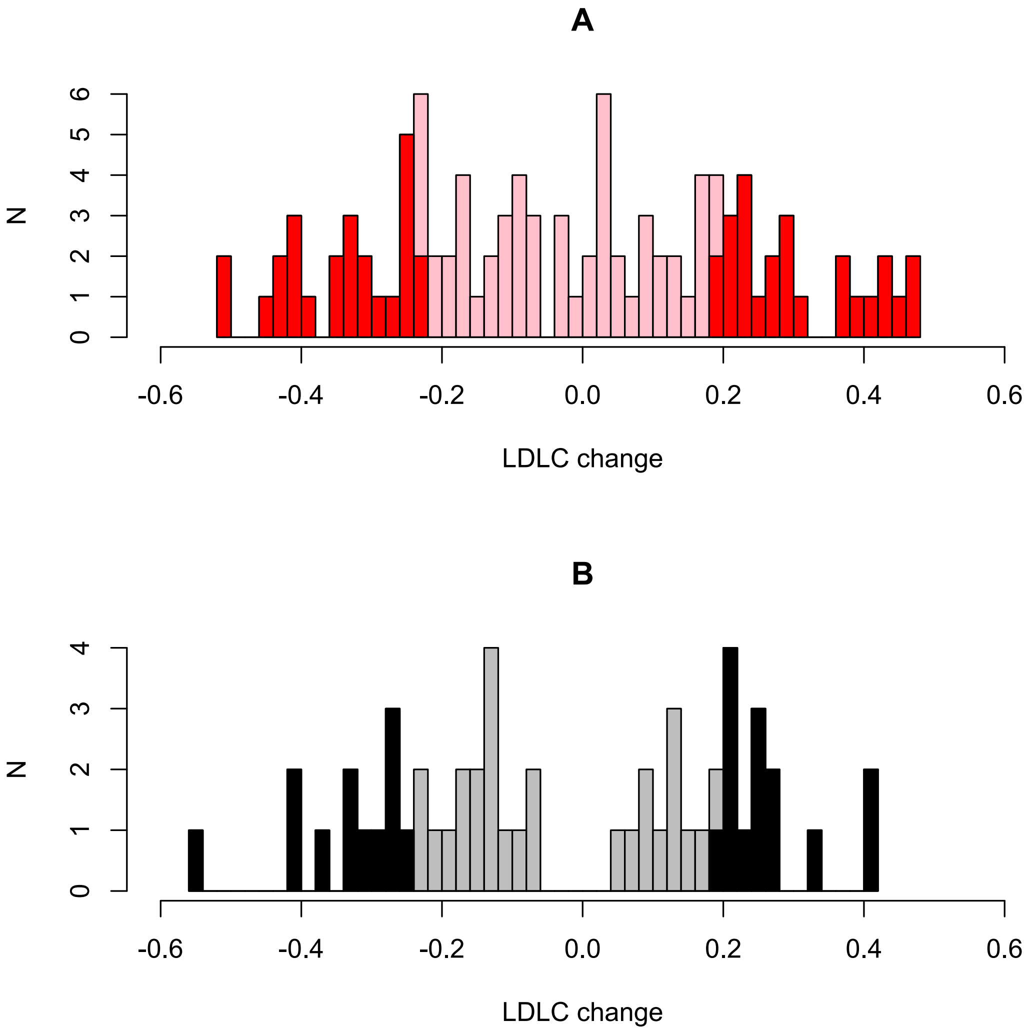

Figure S1. Age, race, and smoking adjusted delta ln LDLC (on simvastatin treatment – baseline) of **(A)** 104 European Americans and **(B)** 53 African Americans from the CAP clinical trial. 50 and 26 extreme responders were color-coded with **(A)** red and **(B)** black.

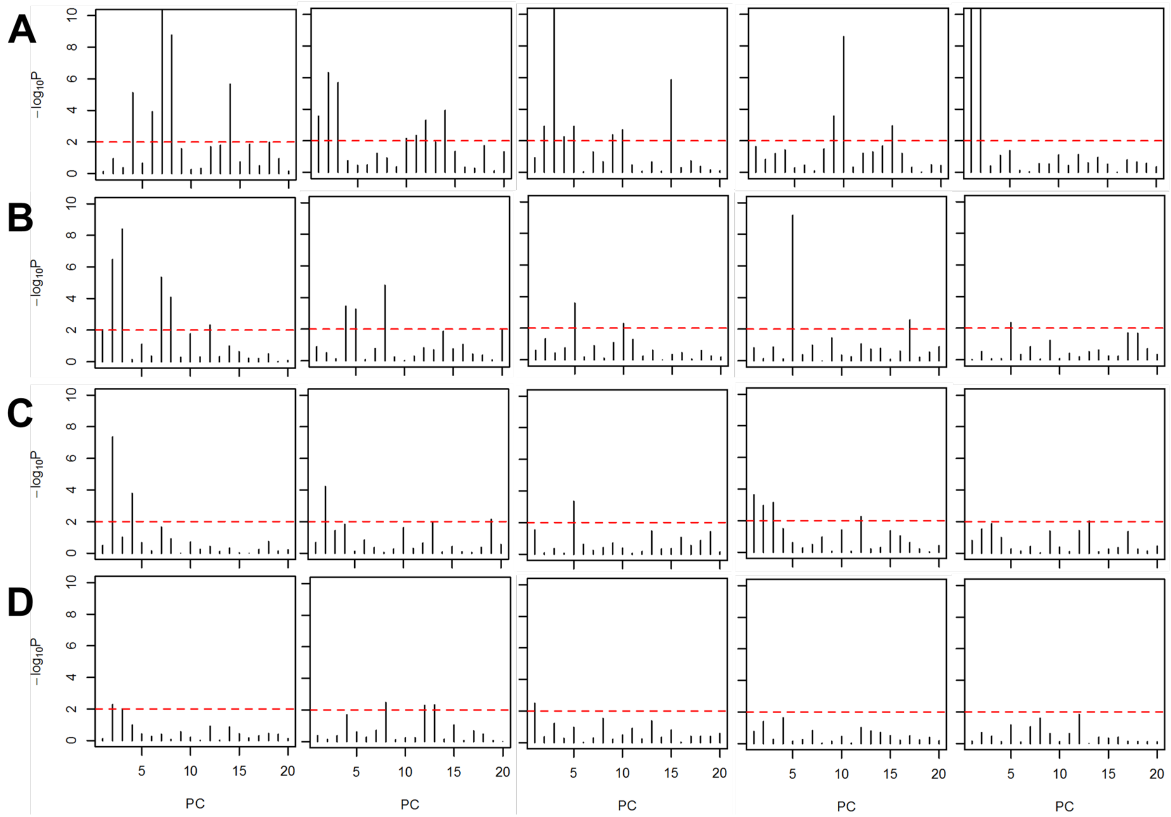

Figure S2. The correlations of five covariates with the 20 PCs derived from (A) non-corrected data, (B) 5 PCs, (C) 10 PCs, and (D) 15 PCs corrected datasets. The covariates are the differences between the statin and sham libraries for total aligned reads (column 1), fraction of ribosomal RNA reads (column 2), fraction of reads falling within Ensemblv67 annotated mRNAs (column 3), fraction of reads aligning to the Ensemblv67 annotated strand (column 4), and 5' to 3' bias (column 5). The significance of correlation is given by $-\log_{10}P$ value and the red dotted line denotes $P = 0.01$.

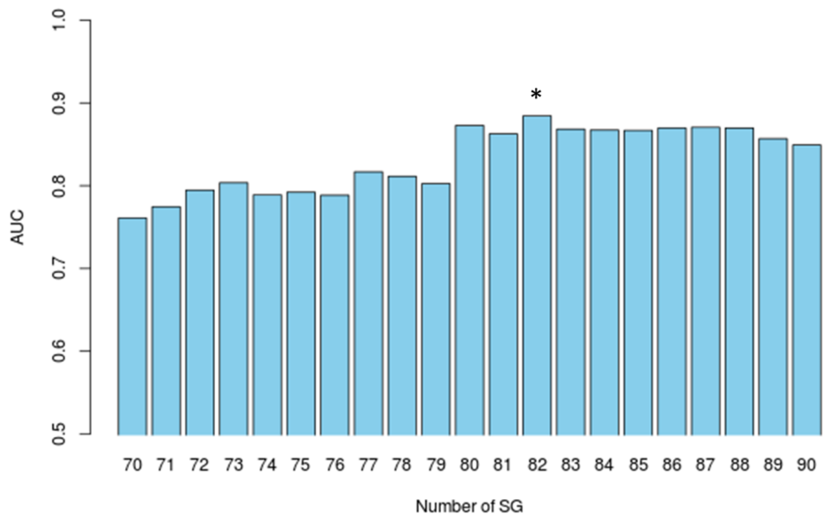

Figure S3. European American signature gene (SG) set prediction performance in 12 African American high and 14 African American low responders as measured by Area under the curve (AUC) values derived from ROC curves. Numbers of signature genes from the 15 PC corrected dataset were varied from 70 to 90 to identify the number of SGs with maximum predictive power. The maximum AUC was achieved with 82 SG, marked by an asterisk.

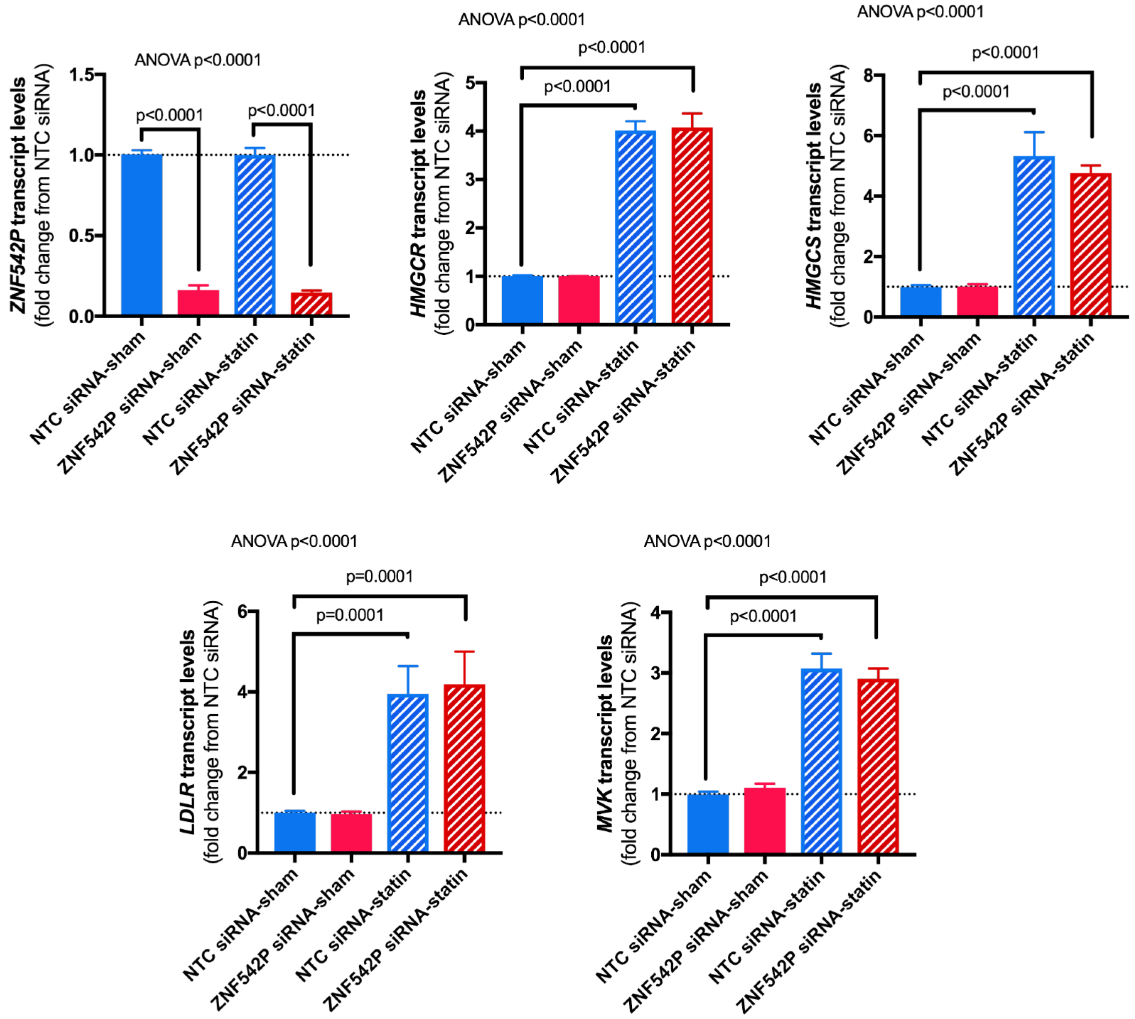

Figure S4. Effect of ZNF542P knock-down on transcript levels. Huh7 cells were transfected with Silence Select siRNAs against *ZNF542P* or a non-targeting control (NTC) using the siPORT transfection reagent. After 24 hours media was supplemented with 2.0uM activated simvastatin (statin) or sham buffer (sham). After and additional 24 hours, cell pellets were collected and gene expression levels were quantified by qPCR using TaqMan Assays, and normalized against *CLPTM.* All qPCR measures were performed in triplicate. Data was normalized to the NTC siRNA sham treated cells, and values shown are mean ± standard error. One-way ANOVA with a Tukey's test for multiple comparisons was used to identify statistically significant differences between treatment groups. Adjusted p-values for multiple testing are shown. N=6-20 replicates per condition.
